# Annotations

**Published:** 1906-05-19

**Authors:** 


### ANNOTATIONS.

### A Peripatetic Hypnotist.

From the columns of the lay Press we learn that a professor in the psychological department of one of the American universities has announced his intention to establish a hospital on what are described as "exceptionally novel lines." After so piquant an advertisement it is disappointing to find that the institution is to be merely an opportunity for the professor to display his powers as a hypnotist in the cure of all cases which he considers to be "supernormal." This term, being interpreted, is found to include cases of "hallucination, secondary personality, psychic epilepsy, neurasthenia, and functional and mental diseases." Of all this we have heard before, not once only but often, and probably if a detailed history of the human race were accessible similar pronouncements could be traced at regular intervals since the days of the Witch of Endor. The peculiar, or possibly peculiar, method which this latest exponent of hypnotism intends to pursue is the practice of what may be termed compulsory therapeutics. He is not to be content with those who will seek succour within the walls of his hospital. On the contrary, it is announced that he will, in the streets, on tramway-cars, and in other public places, seek out suitable patients, and more particularly persons manifestly under the influence of alcohol, and these without let or hindrance he will promptly hypnotise and cure. Not a few drink cures have claimed a capacity for administration without the knowledge of the patient, who, all unconscious, takes the magic drops "in his tea." But there is something weird in the suggestion that the toper or midnight reveller may suddenly, without warning, and while yet in his cups, become whether he will or no, a sober and reformed character under the influence of the passing hypnotist. It is by no means sure that, were this to be realised, the apostle would excite invariable gratitude, and even if he avoids the interference of the police the rage of some disappointed bacchanalian may procure for him the advantage of martyrdom and—advertisement.

### The Spirochæta Pallida.

The causal organism of syphilis, which has proved so long elusive, is gradually being dragged into the open. The spirochæta which Schaudinn first credited with the causation of the disease has received an attention proportionate to its importance, with the result that its presence has been proved to be not invariably in cases of undoubted syphilis. This rather discomfiting fact finds a likely explanation in the theory that the spirochæte form is only a phase in the life-history of the parasite. In the *British Medical Journal* of May 12 is an instructive study from the pen of Dr. Alex. Maclennan. This observer has noted that, while the spirochæta is often not to be found in the discharges from cleaned syphilitic sores, there are constantly present multitudes of minute spherical bodies which he regards as syphilitic organisms in the active stage of their development. Unfortunately the size of these bodies is so minute that detailed investigation of their intimate structure can only be carried out with the help of the highest powers of the microscope. Dr. Maclennan has found that the spirochæte itself is more easily demonstrated in syphilitic discharges after mercurial treatment has been inaugurated than before it. From this circumstance he conjectures that this may prove to be the form assumed by the organism when it commences a struggle for its existence. If this be so the process finds its analogy in the spore-formation which characterises the resting stage of such organisms as the tetanus or anthrax bacilli. In connection with this subject we may refer to a telegram from Paris in a recent issue of the *Times* to the effect that inunction of a mercurial ointment into a wound artificially infected with syphilitic virus is sufficient to prevent infection, provided that the interval between inoculation and inunction does not exceed some twenty hours. While this experiment is of importance as regards prophylaxis, it does not appear to justify the flourish of trumpets which accompanied its publication, for it is almost axiomatic that prompt application of a local antiseptic will prevent a local parasitic infection.

### "To the Mothers of Huddersfield."

There can be no doubt that the great majority of mothers desire to do their best for their offspring. Sympathy for the helpless, an innate sense of responsibility, with perhaps an appreciation of the value of the possession strengthened by the trials of maternity, combine to form a mother's love. Knowledge and wisdom are not, however, always on a level with devotion, while domestic troubles or anxiety as to the necessities of life may absorb attention and partially deaden affection. It is under such circumstances that a knowledge of others being ready to give interest and sympathy may revive the failing spirit. An open letter, with the title "To the Mothers of Huddersfield," has been printed by the Mayor of that borough for general circulation, mentioning methods which have been adopted by the Corporation to aid in preserving the life of babies. Mothers are requested to give notice to the health office in the town of the birth of a baby. One of two lady health visitors—qualified medical women—then call on the mother and give any help or advice that may be necessary. It is impossible, however, for these lady health visitors to visit every house frequently where there is a baby, and therefore the aid of several voluntary lady helpers has been obtained, who will, "so far as they can, justify their name and designation of helpers." The letter draws attention to the wonderful results obtained by means of advice and aid given to mothers in the French village of Villiers le Duc, where no baby has died during a period of ten years. It is wisely pointed out that no such surprising success is to be expected in the thickly-populated borough of Huddersfield, but it is hoped, and no doubt with good reason, that many infant lives may be saved. These efforts of the Corporation of Huddersfield will be watched with interest, and will no doubt stimulate many to follow their praiseworthy example.

